# KLF4 Acts as a wt-CFTR Suppressor through an AKT-Mediated Pathway

**DOI:** 10.3390/cells9071607

**Published:** 2020-07-02

**Authors:** Luis Sousa, Ines Pankonien, Luka A Clarke, Iris Silva, Karl Kunzelmann, Margarida D Amaral

**Affiliations:** 1 BioISI – Biosystems & Integrative Sciences Institute, Faculty of Sciences, University of Lisbon, 1749-016 Lisbon, Portugal; lmdsousa@fc.ul.pt (L.S.); ipankonien@fc.ul.pt (I.P.); laclarke@fc.ul.pt (L.A.C.); iasilva@fc.ul.pt (I.S.); 2 Department of Physiology, University of Regensburg, 93053 Regensburg, Germany; karl.kunzelmann@vkl.uni-regensburg.de

**Keywords:** cystic fibrosis, KLF2, KLF5, epithelial differentiation, AKT signaling, CFTR, GSK3β

## Abstract

Cystic Fibrosis (CF) is caused by >2000 mutations in the CF transmembrane conductance regulator (CFTR) gene, but one mutation—F508del—occurs in ~80% of patients worldwide. Besides its main function as an anion channel, the CFTR protein has been implicated in epithelial differentiation, tissue regeneration, and, when dysfunctional, cancer. However, the mechanisms that regulate such relationships are not fully elucidated. Krüppel-like factors (KLFs) are a family of transcription factors (TFs) playing central roles in development, stem cell differentiation, and proliferation. Herein, we hypothesized that these TFs might have an impact on CFTR expression and function, being its missing link to differentiation. Our results indicate that KLF4 (but not KLF2 nor KLF5) is upregulated in CF vs. non-CF cells and that it negatively regulates wt-CFTR expression and function. Of note, F508del–CFTR expressing cells are insensitive to KLF4 modulation. Next, we investigated which KLF4-related pathways have an effect on CFTR. Our data also show that KLF4 modulates wt-CFTR (but not F508del–CFTR) via both the serine/threonine kinase AKT1 (AKT) and glycogen synthase kinase 3 beta (GSK3β) signaling. While AKT acts positively, GSK3β is a negative regulator of CFTR. This crosstalk between wt-CFTR and KLF4 via AKT/ GSK3β signaling, which is disrupted in CF, constitutes a novel mechanism linking CFTR to the epithelial differentiation.

## 1. Introduction

Cystic Fibrosis (CF) is the most common lethal genetic disease among Caucasians, with a variable geographic prevalence of 1:2500–6000 in Europe, according to the European Cystic Fibrosis Society registry [1]. Over 2000 mutations in the gene encoding the CF transmembrane conductance regulator (CFTR) protein have so far been reported, but the deletion of the phenylalanine at the position 508 (F508del) is by far the most common one, present in at least one allele in ~80% of individuals with CF worldwide. The F508del mutation impairs CFTR protein folding and plasma membrane (PM) trafficking, causing CFTR retention at the level of the endoplasmic reticulum (ER), with only a minimal fraction reaching the PM [2] with decreased function and stability. The association of CFTR to epithelial differentiation has been described in several studies (reviewed recently in [3]).

Being CFTR a chloride/bicarbonate channel, it is not expected to be a direct regulator of differentiation and epithelial regeneration. Therefore, such a role possibly relies on its positive/negative effect on transcriptional factors (TFs) that act at the nuclear level to regulate proliferation and differentiation [4]. Moreover, it was also established that CFTR expression is heavily dependent on a variety of transcriptional factors (TFs) and miRNAs [5]. Among candidate TFs that could link CFTR to epithelial differentiation are the Krüppel-like factors (KLF’s) family members, in particular KLF’s 2, 4, and 5, which are known to regulate those processes [6]. Indeed, one study coupled the regulation of CFTR to KLF4 through a common regulator—miR-145 [7]. Moreover, KLF4 is induced transiently in response to wounding, and this phenomenon is absent in CF airway cells [8]. In parallel, genomic analyses of open chromatin in human tracheal epithelial cells revealed that KLF5 is part of a transcriptional network that represses CFTR gene expression [9,10] and such analyses predicted that in human intestinal organoids, KLF4 was among the top genes expected to be expressed at high levels, followed by CFTR [11]. Remarkably, another study reported that KLF2 is increased by 2.5-fold in CF mouse pre-adipocytes, precluding their differentiation [12], and yet another described that KLF2 expression is lost in CF cells [13]. Moreover, KLF2 has been identified as playing a role in the inflammatory process with a possible impact within the CF context [14].

Altogether, the above studies led us to investigate whether those three KLF family members have an impact on CFTR expression and function.

Our data show that among these, only KLF4 is upregulated in CF vs. non-CF cells in human respiratory epithelial cells. Functional studies show that overexpressing KLF4 has a negative impact on wt-CFTR expression and function, but F508del–CFTR expressing cells are insensitive to KLF4 modulation. In an attempt to investigate the possible pathways linking KLFs to CFTR in both CF and non-CF contexts, we investigated how KLF4-related serine/threonine kinase 1 (AKT) and glycogen synthase kinase 3 beta (GSK3β) pathways affect CFTR. Our data reveal that, while KLF4 modulates CFTR via AKT signaling, GSK3β negative impact on CFTR is somewhat independent of KLF4.

This crosstalk between wt-CFTR and KLF4 via AKT signaling, which is disrupted in CF, constitutes a novel mechanism linking CFTR to the epithelial differentiation.

## 2. Materials and Methods

Detailed descriptions of the methods can be found in the Appendix A.

### 2.1. Human Lungs

Explanted human lungs (wt and F508del-CFTR homozygous) were collected as before [15] and following approval by the hospital Ethics Committee (Ethical code number EK-300/15, date of approval March 4th, 2015).

### 2.2. Chemicals, Antibodies, and Primers

Lists of primary and secondary antibodies used in both Immunofluorescence (IF) and Western Blot (WB) are in Supplementary Tables S1 and S2, respectively. Sequences for primers used in qRT-PCR are in Table S3. siRNAs used are listed in Supplementary Table S4 and inhibitors in Supplementary Table S5.

### 2.3. Cell Lines

CF-relevant immortalized bronchial epithelial cell lines, CFBE41o- (Cystic Fibrosis bronchial epithelial) cells, stably overexpressing wt- and F508del–CFTR [16], were used in this work and grown as previously described [17]. To achieve polarization, cells were seeded on collagen IV pre-coated transwell permeable supports. On the following day, media was changed from 10% to 2% (*v*/*v*) FBS to promote differentiation/polarization. The transepithelial electrical resistance (TEER) was measured regularly. The KLF4 knockout cell lines generated by the CRISPR-Cas9 technique were grown under the same conditions as the other CFBE cells.

For siRNA transfection, cells were transfected in suspension in 24-well plates 24 h after being split. Transfection mixture using Lipofectamine 2000 (Invitrogen, Carlsbad, CA, USA) was prepared, containing 50 nM of siRNA (Supplementary Table S4), according to the manufacturer’s instructions. After transfection, cells were grown in FBS-free media. After 24 h, the media was changed to Eagle’s Minimum Essential Medium (EMEM) supplemented with 5% FBS (*v*/*v*). Seventy-two hours after transfection, cells were harvested. The respective negative controls were used.

Suspension transfection of CFBE41o- cells with plasmids containing GFP tagged KLF4 (or GFP as negative control) was performed in 24 well-plates 24 h after being split, following the manufacturer’s instructions. Twenty-four hours later, the media was changed to EMEM supplemented with 10% (*v*/*v*) FBS. Forty-eight hours post-transfection, the protein extraction was performed. KLF4-GFP used was from Origene (RG206691; Rockville, MD, USA), and the Green Fluorescent Protein (GFP)control used was pEGFP-C2 from Clontech (Mountain View, CA, USA).

### 2.4. KLF4-KO Cells Generation Using CRISPR/Cas9

The Cas9 plasmid was obtained from Addgene (pCas9_GFP, #44719). pCas9_GFP was used with two guide RNAs (1. 5′-GGGGCGGCCGGGAAGCACTG-3′), 2. 5′- GAAACCTTACCACTGTGACT-3′) targeting the genomic region of KLF4, constructed using Invitrogen’s GeneArt^®^ Gene synthesis system. The knockout of KLF4 was carried out using the CRISPR/Cas9 system as previously described [18] using Lipofectamine 2000 (Invitrogen, Carlsbad, CA, USA) for cell transfection. For clone selection, a plasmid containing the hygromycin resistance gene was co-transfected. Cell clones were isolated using clonal discs. Once expanded, genomic DNA from each clone was isolated and amplified by PCR with primers recognizing sequences covering the gRNA targeted region. PCR products were sequenced to identify KLF4-KO clones, which were also confirmed by WB using KLF4 antibody for detection.

### 2.5. RT-qPCR

RT-qPCR was performed as previously described [19]. A list of primers is available in the Supplementary Data.

### 2.6. Biochemical Assays

For co-immunoprecipitation, we used a previously described protocol [20]. Western blot analysis of cell lysates was also previously described [21]. Lists of primary and secondary antibodies used are in Supplementary Tables S1 and S2.

### 2.7. Immunofluorescence Staining (IF)

The IF protocol used has been previously described [19]. Lists of primary and secondary antibodies used are in Supplementary Tables S1 and S2.

### 2.8. Ussing Chamber Experiments

CFBE cells polarized for 7 days were mounted into a micro-Ussing chamber and analyzed under open-circuit conditions at 37 °C, as previously described [19].

### 2.9. Patch-Clamp

For patch-clamping, cells were grown on coverslips and transfected with KLF4-GFP and GFP only as control. The GFP signal allowed the detection of transfected cells. After 48–72 h, the cells were used for patch-clamp recordings in whole-cell configuration, as described before [22].

### 2.10. Statistical Analyses

Data are always presented as mean ± SEM. The Student’s *t*-test for unpaired samples was used for statistical analyses. Prism 6 software (GraphPad, Inc., San Diego, CA, USA) was used for graph design and statistical analyses. Significant differences were defined for *p* ≤ 0.05 and marked with an asterisk. Other trends or tests may be stated in the legend. N = 3 unless stated otherwise in the figure or in its legend.

## 3. Results

### 3.1. KLF4 is Upregulated in CF Native Human Lung and Cell Lines vs. Non-CF

**Figure 1:**
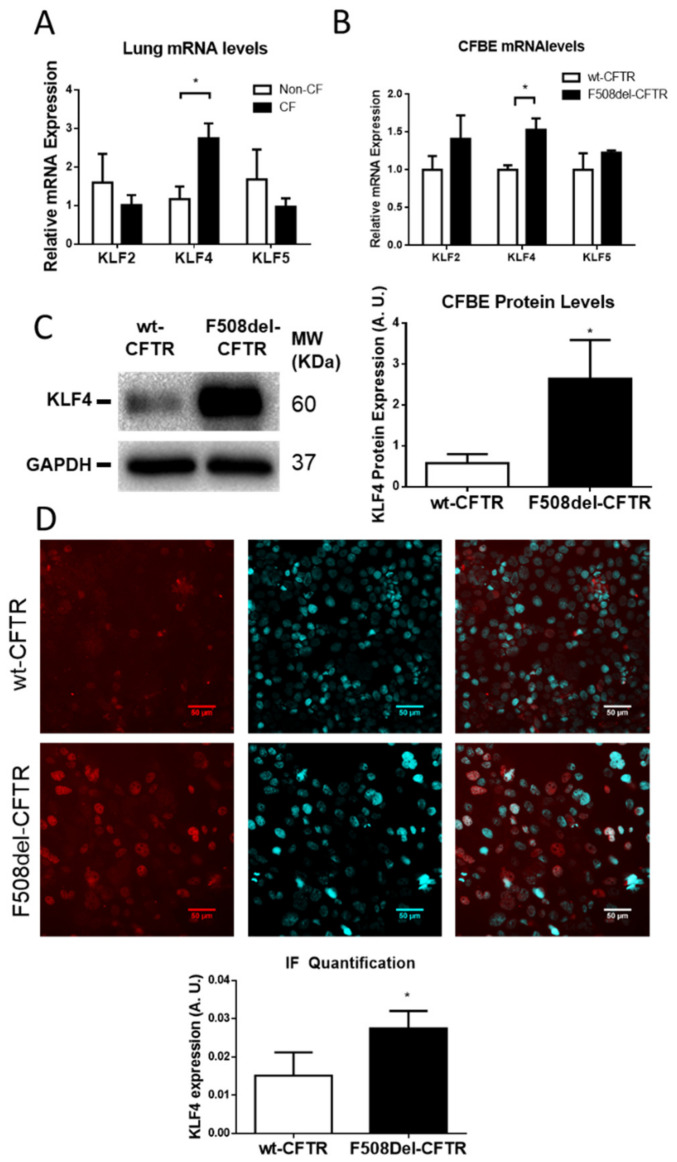
Krüppel-like factor 4 (KLF4) is upregulated in Cystic Fibrosis (CF) native human lung and cell lines. (**A**) KLF2, KLF4, and KLF5 mRNA levels were assessed by RT-qPCR in samples retrieved from lung explant specimens from individuals with CF heterozygous for F508del– CF transmembrane conductance regulator (CFTR) or non-CF controls (n = 4, unpaired *t*-test, *p*-value = 0.02). (**B**) KLF2, KLF4, and KLF5 mRNA levels in CFBE cells expressing wt- or F508del–CFTR assessed by RT-qPCR (n = 3, unpaired *t*-test, *p* < 0.05). (**C**) Representative WB (left) of KLF4 expression in wt- and F508del–CFTR CFBE cells, using Glyceraldehyde 3-Phosphate Dehydrogenase (GAPDH) as loading control and (right) quantification of data in (**A**) in arbitrary units (A.U.) shown as relative expression vs. loading control (n = 3, unpaired *t*-test, *p* < 0.05). (**D**) Representative immunofluorescence staining (IF) images showing KLF4 staining (red, left panels) in wt- and F508del–CFTR expressing CFBE cells, nuclei staining (blue, middle panels) merged images (right panel). Quantification of data below (n = 4, unpaired *t*-test, *p* < 0.05).

To unravel the interplay between the three KLF family members under study (KLF2, 4, and 5) and CFTR in the context of CF, mRNA expression levels of these KLFs were quantified in native human lung specimens from individuals with CF and healthy controls. Data in Figure 1A show that KLF4 expression levels were significantly upregulated (by 2.5-fold) in CF compared to control tissue, whereas no alteration was observed for KLF2 or KLF5 expression levels.

We then evaluated the expression of KLFs in CFBE cells expressing wt- and F508del–CFTR at both RNA and protein levels (Figure 1B,C). In agreement with the data from native lung tissue, both KLF4 mRNA (Figure 1B) and protein (Figure 1C) were found to be significantly upregulated in F508del– vs. wt-CFTR expressing cells, being the levels of KLF4 protein increased by ~5-fold in CF vs. control cells. Immunofluorescence (IF) data, while also confirming higher expression levels of KLF4 in CF vs. control cells, also evidenced that this TF had an almost exclusive nuclear localization in CF cells (Figure 1D). Interestingly, as cell confluency increased, we observed that KLF4 levels steadily increased, coupled with a progressive decrease in the levels of CFTR (Supplementary Figure S1).

### 3.2. KLF4 Downregulation Promotes Expression of wt-CFTR But Not of F508del–CFTR

**Figure 2:**
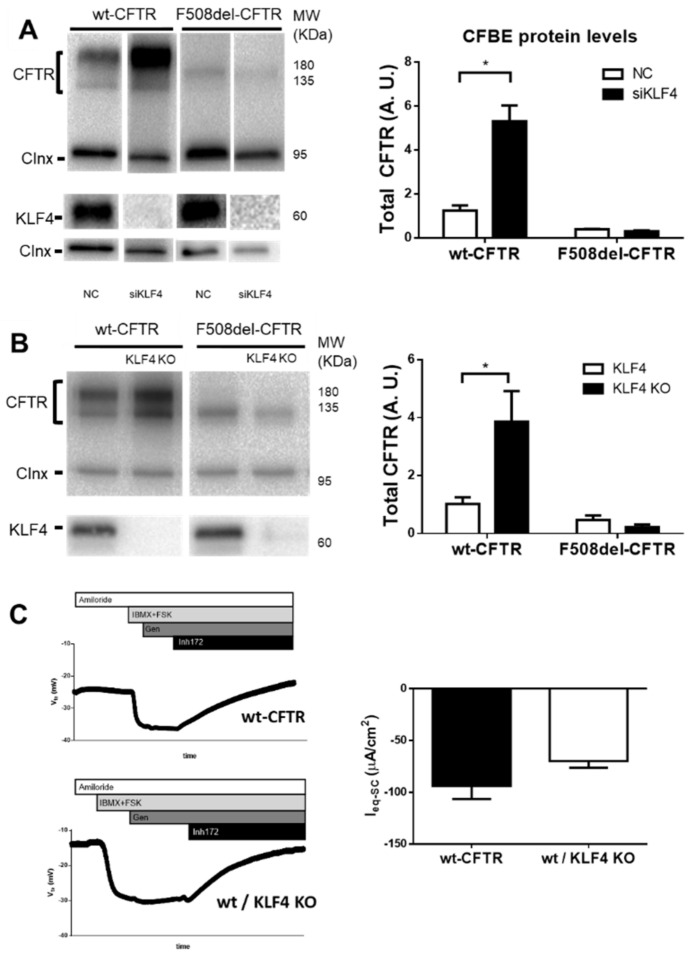
KLF4 knock-down/-out upregulates wt- but not F508del–CFTR. (**A**) Representative WB of KLF4 and CFTR expression in CFBE cells expressing wt- or F508del–CFTR and transfected with either siKLF4 or negative control (NC). Calnexin was used as loading control. Data are normalized to loading control and showed as arbitrary units (A.U.) (n = 3, unpaired *t*-test, *p* < 0.05). (**B**) Representative WB of KLF4 and CFTR expression in wt- and F508del–CFTR CFBE cells and their respective KLF4 KO (KLF4^−/−^). Calnexin was used as loading control. Data are normalized to loading control and showed as arbitrary units (A. U.) (n = 4, unpaired *t*-test, *p* < 0.05). (**C**) Ussing chamber experiments comparing wt-CFTR cells and their KLF4 KO counterparts. Comparable resistances were observed (wt-CFTR cells = 1400 ohm.cm^2^ and wt-CFTR KLF4 KO cells = 1280 ohm.cm^2^) (n = 3, unpaired *t*-test, *p* < 0.05).

To determine whether there was a causal relationship between the observed differences in KLF4 and CFTR expression levels, we then assessed the impact of knocking-down (KD)/out (KO) KLF4 on CFTR expression and function. WB analyses of wt- and F508del–CFTR after KLF4 KD, show distinct effects on wt- and F508del-CFTR: while a dramatic increase resulted in total wt-CFTR levels, no change was observed in F508del-CFTR expression (Figure 2A).

To evaluate possible synergies among KLFs, we then carried out a series of experiments to assess CFTR expression upon KD of KLF2, KLF4, and KLF5 alone or combined (Supplementary Figure S2). Data demonstrated that only KLF4 KD (but neither KD of KLF2 nor KLF5) altered wt-CFTR expression. Noticeably, KD KLF2/5 on top of KLF4 KD seemed to counteract the enhancing effect of KLF KD on CFTR expression by significantly decreasing CFTR levels.

For further validation of the KLF4 effects on CFTR expression, we then evaluated CFTR protein levels in newly generated KLF4 knockout (KLF4-KO) cell lines (clone validation in Supplementary Figure S3). Consistent with KLF4 KD data, KLF4-KO resulted in significantly higher levels of wt-CFTR, but no marked alteration of F508del-CFTR (Figure 2B). Somewhat surprisingly, the increase in CFTR expression resulting from KLF4-KO did not produce a significant effect on CFTR function as analyzed in a Ussing chamber (Figure 2C).

### 3.3. KLF4 Overexpression Decreases Expression and Function of wt-CFTR

**Figure 3:**
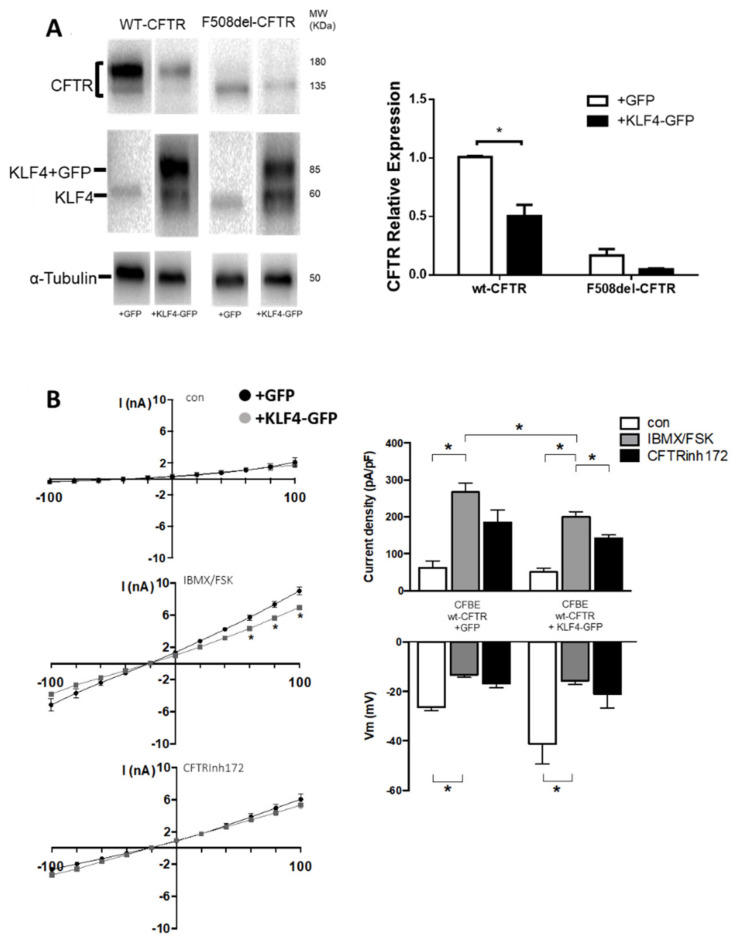
KLF4 overexpression caused a downregulation of wt-CFTR expression and function. (**A**) Transfection with KLF4-GFP was performed, and the effects of KLF4 overexpression on CFTR expression were assessed by WB. Representative WB of KLF4 and CFTR expression in wt- and F508del–CFTR CFBE cells transfected with either negative control (+GFP) or KLF4-GFP (+KLF4-GFP). Beta-tubulin was used as loading control. Data are normalized to loading control and shown as relative expression (vs. wt-CFTR (+GFP)). (n = 3, unpaired *t*-test, *p* < 0.05). (**B**) CFTR chloride currents in CFBE wt cells transfected with GFP (wt) or KLF4-GFP (+KLF4). On the left, current–voltage relationship obtained in CFBE wt-CFTR and CFBE wt-CFTR + KLF4, and effects of IBMX/Fsk (center) and CFTRinh172 (lower). On the right, analysis of CFTR current density (upper) and membrane voltage (lower) before (con), and after application of IBMX/Fsk and CFTRinh172.

To further explore the possible KLF4–CFTR functional relationship, we assessed how KLF4 overexpression affected CFTR expression and function. To this end, we transfected KLF4-GFP or GFP cDNA containing vectors into CFBE cells expressing either wt- or F508del–CFTR. WB data in Figure 3A show that KLF4 overexpression led to a significant decrease in wt-CFTR expression, while no significant impact was observed for F508del–CFTR.

To determine whether KLF4 overexpression also had an impact on wt-CFTR function, we performed patch-clamp experiments (Figure 3B). Data show that although basal currents were not affected by KLF4 overexpression, CFTR currents resulting from IBMX/Forskolin stimulation were significantly lower under KLF4 overexpression in comparison to GFP-transfected cells. By determining the I/V curve under stimulating conditions, a difference in outward currents (Cl^-^ influx) at voltage steps 60, 80, and 100 was also observed, being consistently lower in KLF4 overexpressing vs. control cells.

### 3.4. Characterization of KLF4–CFTR Pathway Crosstalk

Since CFTR is an apical PM protein and KLF4 has mostly a nuclear localization, it is not likely that a direct physical interaction occurs between these two proteins. Still, co-immunoprecipitation (co-IP) was performed to investigate this possibility, and, as expected, it showed no evidence of a CFTR–KLF4 interaction (Supplementary Figure S4).

We thus searched in the proximal CFTR and KLF4 networks for the existence of overlapping pathways. This bioinformatic analysis revealed that epidermal growth factor receptor (EGFR)–AKT and β-catenin (CTNNB) signaling actually link both networks (Supplementary Figure S5), evidencing four common key nodes, namely: AKT (serine/threonine kinase 1, also PKB), EGFR (epidermal growth factor receptor), β-catenin (β-cat), and GSK3β (glycogen synthase kinase 3 beta).

**Figure 4:**
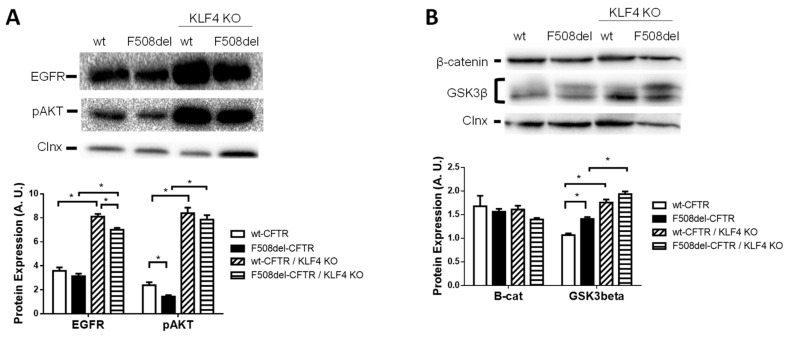
Marked alterations in epidermal growth factor receptor/phosphorylated serine/threonine kinase 1 (EGFR/pAKT) and glycogen synthase kinase 3 beta (GSK3β) signaling pathways were observed. (**A**,**B**) Representative WB of (**A**) EGFR and pAKT, (**B**) beta-catenin and GSK3β expression in wt- and F508del–CFTR CFBE and their respective KLF4 knockouts. Calnexin was used as loading control. Data are normalized to loading control and showed as arbitrary units below (A.U.) (n = 3, unpaired *t*-test, *p* < 0.05).

Accordingly, next, we investigated these four key proteins (AKT, EGFR, β-cat, and GSK3β) in CFBE cells expressing wt- or F508del–CFTR as well as their modulation through KLF4 (Figure 4). AKT was investigated in its phosphorylated (active) form (pAKT). Our results showed that pAKT was downregulated (Figure 4A), and GSK3β was upregulated (Figure 4B) in F508del– vs. wt–CFTR expressing CFBE cells, while the levels of EGFR and β-cat were slightly, albeit not significantly, lower in CF cells (Figure 4A,B, respectively).

To determine the KFL4-dependence of these effects, we investigated these four proteins in KLF4-KO cells expressing wt- or F508del–CFTR (Figure 4). Our data showed that in the absence of KLF4, levels of pAKT, EGFR, and GSK3β (but not β-cat) dramatically increased in both wt- and F508del–CFTR cells. Regarding the differences in CF vs. non-CF cells, while the trend was maintained (lower AKT and EGFR and higher GSK3β in CF cells), only EGFR levels were significantly different between wt- and F508del–CFTR KLF4-KO cells. For GSK3β we noted the appearance of a second band, which was particularly increased in F508del–CFTR KLF4-KO cells (Figure 4B).

**Figure 5:**
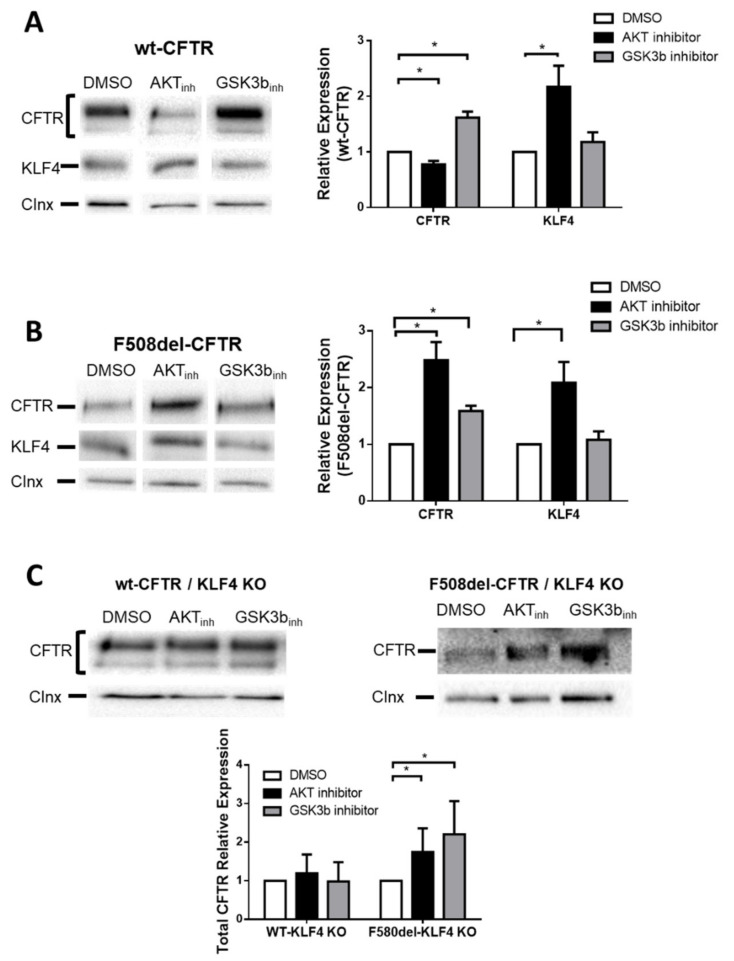
AKT inhibition impacts negatively on wt-CFTR expression and positively on F508del–CFTR, while GSK3β inhibition impacts positively on both wt- and F508del–CFTR. Representative WB of CFTR and KLF4 expression in wt- (**A**) and F508del–CFTR CFBE (**B**) and their respective KO counterparts (**C**) under DMSO or the inhibitors treatments. Calnexin was used as loading control. Data are normalized to loading control and showed as relative expression (vs. DMSO) (n = 3, unpaired *t*-test, *p* < 0.05).

We then tested how modulation of these pathways and their KLF4-dependency affects CFTR expression. To this end, first, we blocked AKT using the chemical inhibitor MK-2206 [23]. Our data from AKT inhibition in wt- and F508del–CFTR expressing cells led to differential effects, both significant (Figure 5): levels of wt-CFTR decreased (Figure 5A), while those of F508del–CFTR (immature form, band B) increased (Figure 5B). Noticeably, by assessing the processing of wt-CFTR (as measured by band C/total CFTR), a decrease in processing of ~10% was found under AKT_inh_. This is suggestive that besides processing itself, other processes, such as recycling, degradation, and PM stability, may be affected by AKT inhibition. In parallel, we observed that under MK-2206, KLF4 levels increased in both wt-CFTR (Figure 5A) and CF cells (Figure 5B). These data suggest that, while KLF4 can be the cause of wt-CFTR downregulation, its effect is not exerted on F508del–CFTR, as above (Figure 2A,B).

Next, we blocked GSK3β with chemical inhibitor TWS119 [24]. In contrast to AKT inhibition, GSK3β inhibition caused the same effect in wt- and F508del–CFTR expressing CFBE cells, leading to increased levels of both normal (Figure 5A) and mutant CFTR (Figure 5B). In parallel, there was no significant change in KLF4 levels in both cell types (Figure 5A,B).

To address whether those observed differences were KLF4-dependent, we then tested the effects of these two chemical inhibitors in KLF4-KO cells expressing either wt- or F508del–CFTR (Figure 5C). Data show that AKT inhibitor no longer decreased wt-CFTR expression in KLF4-KO cells (Figure 5C, left). Similarly, GSK3β inhibitor no longer increased wt-CFTR expression in KLF4-KO cells (Figure 5C, left). These results confirm that the AKT and GSK3β effects on wt-CFTR are KLF4-dependent.

In contrast, the previously observed increases in F508del–CFTR cells under either AKT or GSK3β inhibitors were still present even in the absence of KLF4 (Figure 5C, right), confirming that KLF4 effects are not exerted on F508del–CFTR.

## 4. Discussion

Besides its function as a chloride/bicarbonate channel, CFTR has also been implicated in epithelial differentiation and regeneration [3], being, however, unclear how this occurs mechanistically. Here, we investigated whether the Krüppel-like factors (KLFs), a family of evolutionary conserved zinc finger transcription factors that regulate a variety of biological processes including proliferation, differentiation, and apoptosis [6], have an impact on CFTR expression and function. Among these TFs, we selected KLF2, KLF4, and KLF5 because of previous reports relating these TFs to CFTR [7,8,9,10,11,12,13,14] and for their reported role on differentiation [6].

Our data indicated that among the three KLFs tested, only KLF4 is altered (upregulated) in CF, both in lung tissue and in CF cell lines vs. controls. Furthermore, downregulation of the three KLF’s showed that only KLF4 KD by itself was able to exert a significant decrease in wt-CFTR (but not F508del) expression, indicating a specific impact of KLF4 on normal CFTR. However, we also observed that different KD combinations (namely siKLF2/siKLF4, siKLF4/siKLF5, and siKLF2/siKLF4/siKLF5) promoted a significant decrease in CFTR expression. This may indicate that there is some compensatory mechanism between KLFs, as well as some degree of redundancy. In fact, KLF2 and KLF4 partial redundancy has been previously reported [25], although our data did not confirm this. However, KLF5 and KLF2 concomitant knockdown display no major impact on wt-CFTR levels, suggesting that KLF5 or KLF2 KD require KLF4 repression to produce the observed wt-CFTR downregulation. Interestingly, previous studies have indicated KLF5 as a repressor of CFTR [10], an effect that we could not observe here. Since KLF4 and KLF5 regulate each other [26], it is possible that the observed effect results from the interplay of these two TFs.

Moreover, our data also showed that KLF4 modulation impacts CFTR expression levels and activity. The KLF4 KD/KO experiments lead to increased levels of wt-CFTR. However, this was not accompanied by a corresponding increase in CFTR activity as assessed by Ussing chamber experiments, which we attribute to a technical limitation possibly because CFTR levels activity measured were already close to their maximum. It is also possible that Fsk-stimulated activity of wt-CFTR can still be maximally activated, namely by phosphorylation [27,28]. Thus, depending on the phosphorylation state of the cell, there is still room to maximize Fsk-stimulated activity of wt-CFTR further. However, in converse experiments (i.e., under KLF4 overexpression), we did observe a marked decrease both in the wt-CFTR expression and activity. This is further validated by our confluency assays, in which increased levels of confluency were coupled to increased KLF4 and decreased wt-CFTR expression.

Interestingly, the concept that KLF4 activity is highly context-dependent [29] also emerges from our data. In fact, F508del–CFTR CFBE cells appeared to be refractory to KLF4 modulation since the ability of the latter to negatively regulate CFTR was lost. It is possible that the intrinsic instability of F508del-CFTR protein [30] could mask the enhancement of immature F508del–CFTR protein by KLF4. In parallel, since it is established that F508del- and wt-CFTR have different interactomes [31,32,33,34,35], F508del–CFTR as an immature/unstable protein does not establish the required interactions for KLF4 to exert its signaling as an F508del–CFTR modulator. Moreover, alterations in intracellular pH caused by defective CFTR may also have an impact on the signaling exerted by KLF4 [36] In fact, KLF4 KD/KO and overexpression experiments showed no major impact on F508del–CFTR expression. Dysfunctional KLF4 in CF cells may be the cause of its observed overexpression in CF, possibly via compensatory mechanisms.

We speculate that this may be due to the fact that CF cells display a partial EMT/cancer-like phenotype [3], which leads to altered signaling pathways, namely those linking KLF4 to CFTR. Indeed, KLF4 regulates gene expression through transcriptional activation or repression via either DNA binding or protein-to-protein interactions, and thus the outcome of KLF4-mediated regulation largely depends on the cellular context, e.g., the presence of oncogenic drivers among other factors [29].

The negative regulation of wt-CFTR (but not F508del–CFTR) by KLF4 led us to consider that KLF4 may play a particularly interesting role in non-CF cells and prompted further mechanistic investigation.

Although the CFTR gene possesses several major enhancer/promoter regions potentially binding several TFs [37], KLF4 (nor KLF2, KLF5) is not predicted to bind these regions. In fact, CFTR is not among its KLF4-transcribed genes [25]. We also did not find a direct interaction between these two proteins. Therefore, the KLF4-CFTR crosstalk must be mediated by signaling pathways and/or interactors.

Bioinformatic network analysis suggested two plausible pathways linking KLF4 and CFTR, namely those involving GSK3β/β-catenin and EGFR/AKT.

Our data indicated that pAKT is downregulated, and GSK3β is upregulated in F508del–CFTR CFBE cells, while EGFR and total β-cat levels are unchanged. Although observed in previous studies, our data did not indicate alterations in the β-catenin signaling in the CF context. We speculate that this may be due to the fact that those studies used different cell models/organisms [36,38,39,40]. It is also possible that the levels of active β-catenin were altered because we only measured total β-catenin. Interestingly, GSK3β upregulation in CF cells occurred with the concomitant appearance of a second band, which may correspond to an isoform implicated in other diseases [41]. Moreover, our results also indicated that KLF4 appears to act as a repressor of pAKT, EGFR, and GSK3β (but not β-cat), as in KLF4-KO cells there was a marked increase in the levels of these proteins. To pinpoint the effects of AKT and GSK3β on CFTR, we chemically inhibited these two proteins. Under AKT inhibition (MK-2206), we observed a differential effect on wt- and F508del–CFTR again: while wt-CFTR levels markedly decreased, those of F508del–CFTR significantly increased. Interestingly, KLF4 levels increased in the wt-CFTR expressing cells but remained unchanged in CF cells.

**Figure 6:**
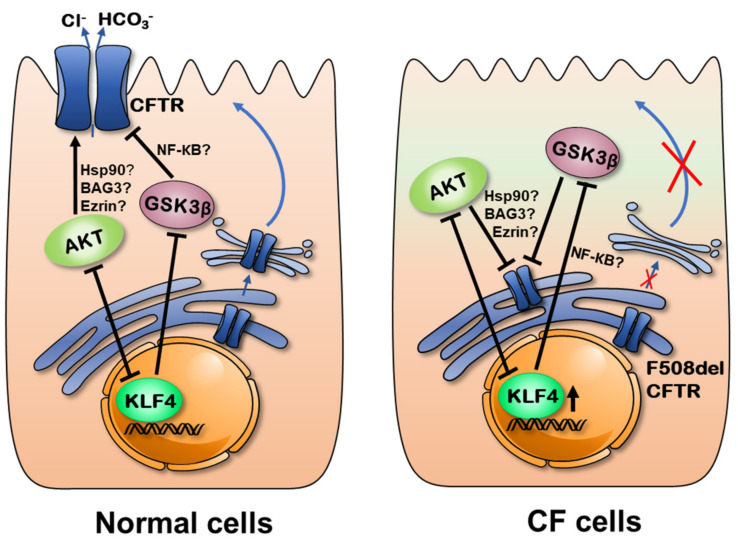
KLF4 acts as a negative regulator of wt-CFTR but has its function disrupted in the F508del–CFTR cells. Our data suggest that the AKT pathway is a positive regulator of wt-CFTR while being a negative regulator of F508del–CFTR in a way that is possibly at least partially mediated by KLF4, which in turn seems to be negatively regulated by AKT signaling in wt-CFTR cells. GSK3β, on the other hand, seems to be a negative regulator of CFTR, which is KLF4-dependent in wt-CFTR and KLF4-independent in F508del–CFTR expressing cells.

Altogether, these data strongly suggest that the effect of AKT on wt-CFTR is mediated by KLF4 (Figure 6, left): when AKT was present, KLF4 levels were kept low, and wt-CFTR was normally expressed. However, upon AKT inhibition, KLF4 was derepressed, downregulating CFTR. In contrast, AKT seems to act as an active repressor of F508del–CFTR regardless of KLF4 (Figure 6, right). Indeed, despite the increase in KLF4 by AKT inhibition, F508del–CFTR was still upregulated. This effect of AKT modulation on F508del–CFTR has been previously shown [42]. Moreover, another study targeting the PI3K/Akt/mTOR signaling pathway in CF also showed that inhibition of AKT using MK-2206 increased stability and expression of mutant CFTR and that this effect may possibly be mediated by BAG3 [23]. Another interesting report established the connection between AKT and CFTR through ezrin [43], a known stabilizer of CFTR at the PM [44].

The data shown here for KLF4-KO cells, showing that the AKT inhibitor no longer affected wt-CFTR but still affected F508del–CFTR, imply that the AKT signaling impact on wt-CFTR is KLF4-dependent, but this dependency is lost in CF cells (Figure 6). Noteworthy, KLF4 may have opposing effects on AKT activity, depending on the malignancy levels [45]. This finding may be relevant for the present data if we consider that CF cells display a more cancer-like phenotype [3].

Using the GSK3β inhibitor, we observed an increase in both wt- and F508del–CFTR expression, but with no change in KLF4 levels. Other authors found that GSK3β inhibition rescues F508del–CFTR [46], a finding that we did not detect here, despite the increased levels of band B in F508del–CFTR. Our data suggest that GSK3β acts as a repressor of CFTR (both normal and mutant) and that this effect is not KLF4-independent in wt-CFTR cells, while this seems to be the case in F508del–CFTR cells. However, in KLF4-KO cells, the effect disappeared on wt-CFTR (while remaining on F508del–CFTR), leading us to envisage that GSK3β is also impacted by KLF4, but not vice-versa (Figure 6). Interestingly, KLF4 is a known promotor of Cadherin 3 (CDH3), and GSK3β is a downstream effector of CHD3 [47]. Accordingly, it is plausible that GSK3β levels are affected by KLF4 but not the opposite. In certain particular contexts, such as differentiation, however, GSK3β is required for transient KLF4 expression [48]. For β-catenin, described as a positive regulator of CFTR [38], we could not find a dependence on KLF4 for its effects on CFTR. One possibility is that NF-kB is a mediator of this process [40].

Taking our data globally, we propose that AKT is a positive regulator of wt-CFTR, dependent on KLF4 and that KLF4 may negatively affect wt-CFTR via Akt repression (Figure 6). Possible mediators of this pathway include Hsp90, Ezrin, and BAG3. However, KLF4 has this regulatory role disrupted towards mutant CFTR.

GSK3β, in turn, is a negative regulator of CFTR (both wt and mutant), but only dependent on KLF4 in non-CF cells (Figure 6). Multiple connections between KLF4, AKT, and GSK3β are possible.

Altogether, these data show that KLF4 acts as a negative regulator of wt-CFTR expression and function, but its effects are not exerted on F508del–CFTR. This may be due to the fact that CF cells display a partial EMT/cancer-like phenotype [3] and that CFTR has been proposed to act as a tumor suppressor [49]. This should lead to altered signaling pathways, namely those linking KLF4 to CFTR. Thus, further studies are required to unravel the interplay between these factors and CFTR completely.

By establishing a relationship between CFTR and the AKT/GSK3β pathways and KLF4, all related to differentiation, this work also opens new avenues for CF therapy. Since our data suggest that targeting AKT and GSK3β may increase levels of immature F508del–CFTR, this may be a way to enhance the effect of other therapies, i.e., corrector drugs, that rescue F508del–CFTR. Noticeably, the therapeutic potential of several inhibitors of the AKT and GSK3β signaling pathways have been extensively studied in the context of cancer [50,51]. Interestingly, individuals with CF were also shown to have an increased risk of cancer [52,53,54]. So, these may be safe options worth exploring in further detail. Moreover, considering KLF4, a key factor in differentiation [26], modulation of its downstream effectors may be a way to partially correct the underlying differentiation defect observed in CF [55].

## Supplementary Materials

The following are available online at https://www.mdpi.com/2073-4409/9/7/1607/s1, Figure S1: Confluency levels influence the expression of CFTR and KLF4. Figure S2: Different combinations of siRNAs for KLF2, KLF4 and KLF5 were tested. Figure S3: KLF4 KO validation. Figure S4: Co-immunoprecipitation. Figure S5: Major signaling pathways connecting CFTR to KLF4. Table S1: Primary antibodies used. Table S2: Secondary antibodies used. Table S3: Description of the primers used in qRT-PCR and for gDNA amplification for KLF4 KO validation through genomic sequencing. Table S4: Description of the siRNAs used. Table S5: Description of the used inhibitors.

## Appendix A. Extended Methodology

### Appendix A.1. Cell Lines and Growth Conditions

CF-relevant immortalized bronchial epithelial cell lines, CFBE41o- (Cystic Fibrosis bronchial epithelial) cells, stably overexpressing wt- and F508del–CFTR, were used in this work. CFBE cells were grown in minimum essential medium Eagle (MEM) with Earl salts and L-glutamine (10-010-CVR; Corning, NY, USA) supplemented with 10% (*v*/*v*) Fetal bovine serum (FBS) (10270; Gibco, Waltham, MA, USA) and puromycin (P8833; Sigma–Aldrich, Taufkirchen, Germany) at 2.5 μg/mL for selection. To achieve polarization, cells were seeded on collagen IV pre-coated Transwell permeable supports at a density of 1.25, 2.5, or 10 × 10^5^ cells, depending on the diameter of the filter (6.5 mm, 12 mm, or 24 mm insert, 3470, 3460, and 3450, respectively, Corning, NY, USA). On the following day, media was changed from 10% to 2% (*v*/*v*) FBS to promote differentiation/polarization. The transepithelial electrical resistance (TEER) was measured regularly. The KLF4 knockout cell lines generated by the CRISPR-Cas9 technique were grown under the same conditions as the other CFBE cells. All cells were grown at 37 °C, 5% CO_2_.

### Appendix A.2. Treatment with Compounds

CFBE cells were seeded (100,000 cells) in a P-24 well plate and allowed to grow until fully confluent (24 h). Then, they were treated with AKT inhibitor MK-2206 (S1078; Selleckchem, Houston, TX, USA) and GSK3beta inhibitor TWS119 (S1590; Selleckchem, Houston, TX, USA) for 48 h at a working concentration of 1 µM. Inhibitors were diluted in DMSO at a stock concentration of 40 mM, and DMSO was, therefore, used as a negative control.

### Appendix A.3. siRNA Transfection

For siRNA transfection, 75,000 cells were transfected in suspension in 24-well plates 24 h after being split. Transfection mixture using Lipofectamine 2000 (1 mg/mL, #11668019; Invitrogen, Carlsbad, CA, USA) was prepared, containing 50 nM of siRNA (Table S 4) and 3 ng Lipofectamine to a final volume of 400 μL OptiMEM:EMEM (1:3), according to the manufacturer’s instructions. After transfection, cells were grown in FBS-free media. After 24 h, the media was changed to MEM supplemented with 5% FBS (v/v). Seventy-two hours after transfection, cells were harvested. The respective negative controls were used (Ambion, Austin, TX, USA or Dharmacon, Lafayette, CO, USA).

### Appendix A.4. cDNA Transfection

Suspension transfection of CFBE wt-and F508del–CFTR cells with plasmids containing GFP tagged KLF4 (or GFP for negative control) was performed in 24 well-plates 24 h after being split. Two transfection mixtures were prepared, one with 3 ng Lipofectamine^®^ 2000 (1 mg/mL, #11668019; Invitrogen, Carlsbad, CA, USA) in 50 µL of OptiMEM and one with 1500 ng cDNA in 50 μL of OptiMEM, according to the manufacturer instructions. These two were allowed to incubate for 20 min and then added dropwise to the well containing 300 µL of minimum essential medium Eagle (EMEM), 2% FBS, and 75,000 cells in suspension. Twenty-four hours later, media was changed to EMEM supplemented with 10% (*v*/*v*) FBS. Forty-eight hours post transfection, the protein extraction was performed. KLF4-GFP used was from Origene (RG206691; Rockville, MD, USA), and the GFP control used was pEGFP-C2 from Clontech (Mountain View, CA, USA.

### Appendix A.5. KLF4 KO Cell Generation

The Cas9 plasmid was obtained from Addgene (pCas9_GFP, #44719). pCas9_GFP was used with two guide RNAs (1. 5′-GGGGCGGCCGGGAAGCACTG-3′), 2. 5′- GAAACCTTACCACTGTGACT-3′) targeting the genomic region of KLF4, constructed using the Invitrogen’s GeneArt^®^ Gene synthesis system. The knockout of KLF4 was carried out using the CRISPR/Cas9 system, as previously described [18], using Lipofectamine 2000 (Invitrogen, Carlsbad, USA) for cell transfection. For clone selection, a plasmid containing the hygromycin resistance gene was co-transfected. Cell clones were isolated using clonal discs. Once expanded, genomic DNA from each clone was isolated and amplified by PCR with primers recognizing sequences covering the gRNA targeted regions. PCR products were sequenced to identify KLF4 KO clones, which were also confirmed by WB using KLF4 antibody for detection.

### Appendix A.6. RNA Extraction, Reverse Transcription, and RT-qPCR

Total RNA was extracted from human CF (F508del/F508del) and non CF lung samples using the Direct-zol RNA Miniprep Plus kit (Zymo Research, Irvine, CA, USA), and reverse transcription of cDNA was then performed using 100 ng of each RNA sample, M-MuLV Reverse Transcriptase (NZYtech, Lisbon, Portugal), and random primers. A similar protocol was used for CFBE cells, using for RNA extraction the NZY total RNA isolation kit (MB13402; NZYtech, Lisbon, Portugal), according to the protocol provided.

Then, a mix containing forward and reverse primers, cDNA (5 ng), and 1× Evagreen SsoFast PCR reagent (172-5204; Bio-Rad, Hercules, CA, USA) was used along with a Bio-Rad CFX96 system. Bio-Rad CFX Manager 2.0 software (1845000; Bio-Rad, Hercules, CA, USA) was used for analysis. A standard cycle protocol was used for PCR amplification (1 min at 95 °C followed by 40 cycles of 10 sec at 95 °C and 30 sec at 60 °C).

Technical duplicates were used in amplification, melt curves were examined to confirm the amplification of specific products, and negative controls were confirmed to be free of amplification after 40 PCR cycles. Mean relative levels of expression were calculated for the target genes using the DDCT method, where Fold Change = 2(−ΔΔCT), using mean levels of expression in non CF samples as the baseline (or wt-CFTR cells).

### Appendix A.7. Protein Extraction

For protein extraction cells were washed three times with 1× PBS and lysed in 1× sample buffer (SB) (2× SB –Tris-HCl (Sigma, Taufkirchen, Germany) 62.5 mM, pH 6.8, SDS 3% (15553; Gibco, Waltham, MA, USA), glycerol 20% (92025; Sigma, Taufkirchen, Germany), Bromophenol Blue 0.02% (*w*/*v*), DTT 100 mM (Sigma, Taufkirchen, Germany) supplemented with protease inhibitor cocktail (11697498001; Roche, Mannheim, Germany), 25 U Benzonase (#E1014-25G; Sigma-Aldrich, Taufkirchen, Germany) and 3.125 mM of MgCl2 (Merck, 105833). Lysates were prepared by repeated pipetting and then collected.

### Appendix A.8. Protein Quantification Assay

The Bio-rad protein assay (5000006; Bio-Rad, Hercules, CA, USA) was used to quantify protein extracts. This method is based on the Bradford method. Briefly, the reagent was diluted in water (20:80) a curve was made using different concentrations of the BSA standard (Bio-Rad, Hercules, CA, USA), applying 0–20 µL of the standard into 1000–980 µL of the reagent. Ten microliters of the samples were applied in 990 µL of the reagent. All of these were incubated for 5 min at RT and assessed in a spectrophotometer by measuring the absorbance at 595 nm. Standards were used to create a linear standard curve and the concentration of the samples calculated by plotting the results against the standard curve.

### Appendix A.9. Western Blot

Twenty-five to forty micrograms of protein were loaded onto polyacrylamide gels (4% for stacking and 7% or 10% for resolving gels) to perform SDS/PAGE. Transfer onto polyvinylidene difluoride (PVDF) membranes (IPVH00010; Merck Millipore, Burlington, MA, USA) was performed using a wet-transfer system. The membranes were blocked for 1 h with 5% (*w*/*v*) non-fat milk (NFM) in PBS supplemented with Tween 20 (BP337-100; Fisher Scientific, Hampton, NH, USA). This was followed by incubation with the primary antibody overnight at 4 °C, with gentle shaking. Horseradish peroxidase (HRP)-conjugated secondary antibodies were applied for 1 h at RT. All the antibodies were diluted in the blocking solution. Membrane luminescence was detected on a Chemidoc XRS+ system (170-8265; Bio-Rad, Hercules, CA, USA). Quantification of band intensity was performed using the Image Lab software (170-9690; Bio-Rad, Hercules, CA, USA), which integrates peak area. All measurements were normalized against loading controls (calnexin-Clnx, vinculin, tubulin, or GAPDH). A list of primary and secondary antibodies can be found in Supplementary Table S1 and S2.

### Appendix A.10. Co-Immunoprecipitation

CFBE wt-CFTR and F508del–CFTR cells were lysed at 4 °C with PD buffer (50 mM Tris-HCl, 0.1 mM NaCl, 1% (*v*/*v*) NP40, 10% (*v*/*v*) glycerol, pH 7.5) supplemented with protease inhibitor cocktail (Roche, Mannheim, Germany). Lysates were centrifuged. Pellets were discarded, and the supernatants were pre-cleared through incubation with Protein-G agarose beads (Invitrogen, Carlsbad, CA, USA). The supernatants were then incubated overnight with the appropriate antibody, either anti-CFTR or anti-KLF4, at 4 °C. For control reactions, no antibody was added. Beads were washed three times with wash buffer (Tris-HCl 0.1 M, NaCl 0.3 mM, Triton X-100 1% (*v*/*v*), pH 7.5), followed by elution with 1× sample buffer and further separation on SDS-PAGE and Western blot analysis.

### Appendix A.11. Immunofluorescence Assay

CFBE cells were grown in glass coverslips and then fixed with PFA (104003; Merck Millipore, Burlington, MA, USA) 4% (*v*/*v*), permeabilized with triton X-100 (17-1315-01; Amersham Biosciences, Little Chalfont, UK) 0.5% (*v*/*v*), and blocked with bovine serum albumin (BSA) 1% (*w*/*v*). Cells were then incubated for 2 h with the primary antibody at room temperature, after which a mix of the secondary antibodies and nuclear dye (4 μg/mL, Methyl Green, Sigma–Aldrich, 67060 or Hoechst 33258 (1 μg/mL, 94403; Sigma–Aldrich, Taufkirchen, Germany) was applied for 1 h at RT. Coverslips were then mounted in a mix of N-propylgallate (P3130; Sigma–Aldrich, Taufkirchen, Germany) and glycerol for microscopy (104,095; Merck, Darmstadt, Germany). Imaging was performed with a Leica 6500B microscope. Software used for the acquisition was Leica’s LAS x, and image processing was performed on ImageJ FIJI.

### Appendix A.12. Ussing Chamber Experiments

CFBE cells polarized (as measured by a TEER > 500 ohm.cm2) for 7 days were mounted into a micro-Ussing chamber and analyzed under open-circuit conditions at 37 °C. Apical and basolateral sides were continuously perfused with ringer solutions containing 30 and 145 mM Cl—concentrations (pH 7.4), respectively. After an equilibrium period, 30 μM amiloride (A7410; Sigma–Aldrich, Taufkirchen, Germany) was added apically to block ENaC. Subsequently, the cyclic adenosine 3′-5′ monophosphate (cAMP) agonist, Forskolin (Fsk- 2 μM), the CFTR potentiator Gen (50 μM), and the CFTR channel blocker Inh172 (30 μM) were added sequentially. Values for transepithelial voltages (Vte) were referenced to the basal surface of the epithelium. Transepithelial resistance (Rte) was determined by applying short current pulses (1 s) of 0.5 μA (5-s period). The equivalent short circuit (Ieq-sc) was calculated according to Ohm’s law (Ieq-sc = Vte/Rte).

### Appendix A.13. Patch-Clamp

For patch-clamping, cells were grown on coverslips and transfected with KLF4-GFP and GFP plasmid only as control. The GFP signal allowed the detection of transfected cells. After 48–72 h, the cells were used for measurements. Mounted in a perfused bath on the stage of an inverted microscope, the cells were perfused continuously and held at 37 °C via a water tubing system. Patch-clamp recordings were performed in whole-cell configuration. A glass microelectrode was filled with an intracellular cell-like solution and attached to the cell surface, which led to a high resistance seal formed between the pipette and the cell when applying a slight negative pressure. To achieve the whole-cell configuration, the patch was disrupted by a hard suction, and whole-cell currents were measured using a computer-controlled amplifier, which was connected to the microelectrode. Membrane voltages (V_m_) from -100 to 100 mV were clamped in 20 mV steps.

### Appendix A.14. Statistical Analyses

Data are always presented as mean ± SEM. The Student’s *t*-test for unpaired samples was used for statistical analyses. Prism 6 software (GraphPad, Inc., San Diego, CA, USA) was used for graph design and statistical analyses. Significant differences were defined for *p* ≤ 0.05 and marked with an asterisk. Other trends or tests may be stated in the legend. N = 3 unless stated otherwise in the figure or in its legend. On Western blots, all images compared come from the same blot.
