# Supplementary figures and images for: KLF4 Acts as a wt-CFTR Suppressor through an AKT-Mediated Pathway
Source: Cells. 2020 Jul 2;9(7):1607. doi: 10.3390/cells9071607 (PMC7408019; doi:10.3390/cells9071607)

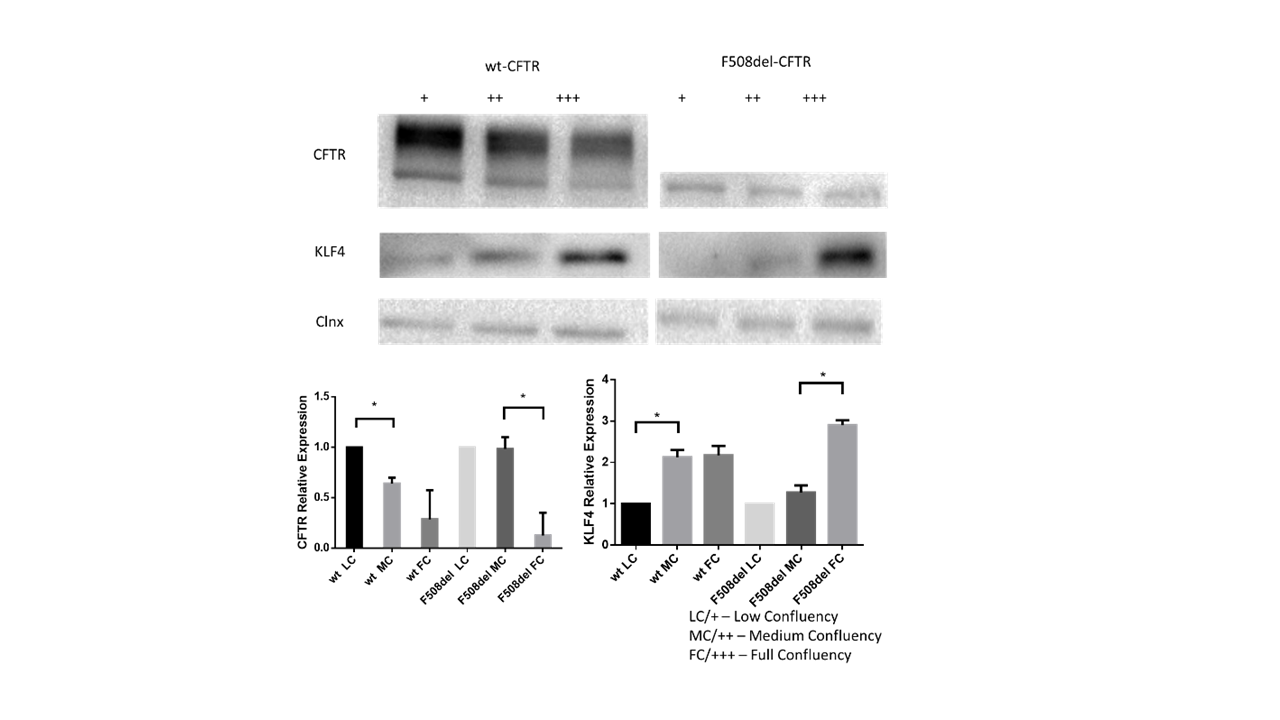

Supplement: Supplementary file 1 [file cells-09-01607-s001.zip › Slide1.PNG]

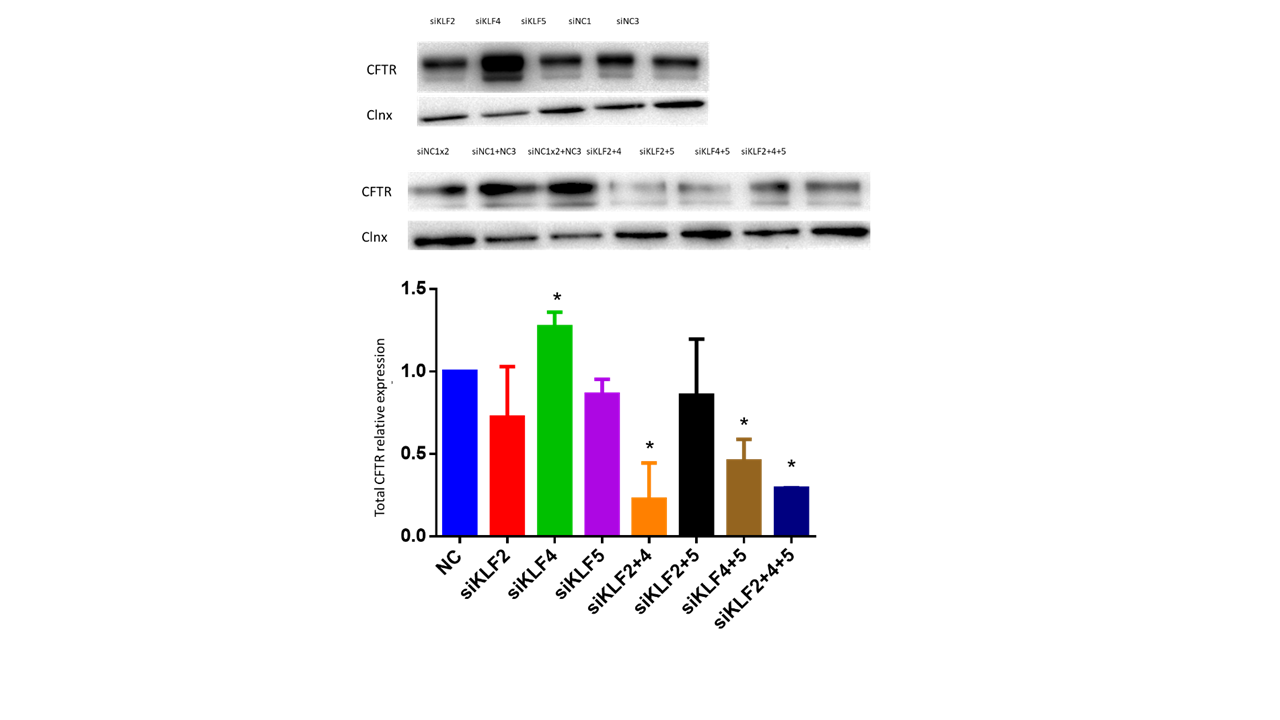

Supplement: Supplementary file 1 [file cells-09-01607-s001.zip › Slide2.PNG]

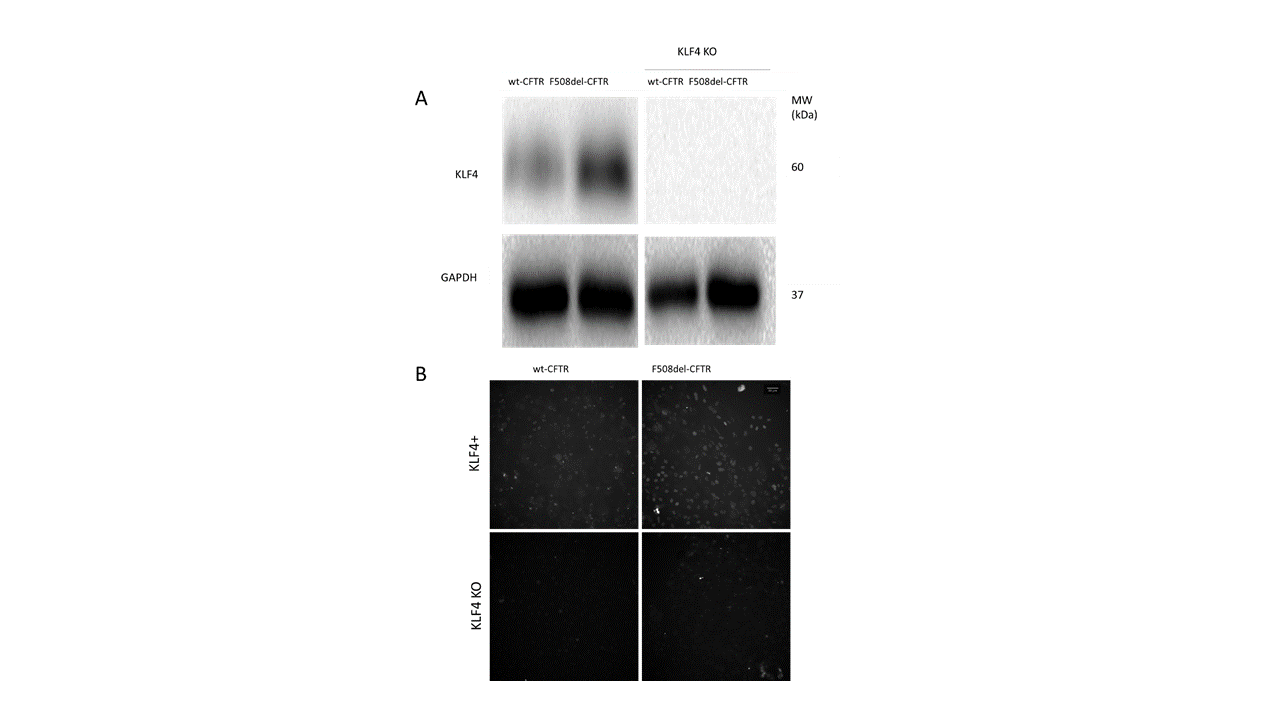

Supplement: Supplementary file 1 [file cells-09-01607-s001.zip › Slide3.PNG]

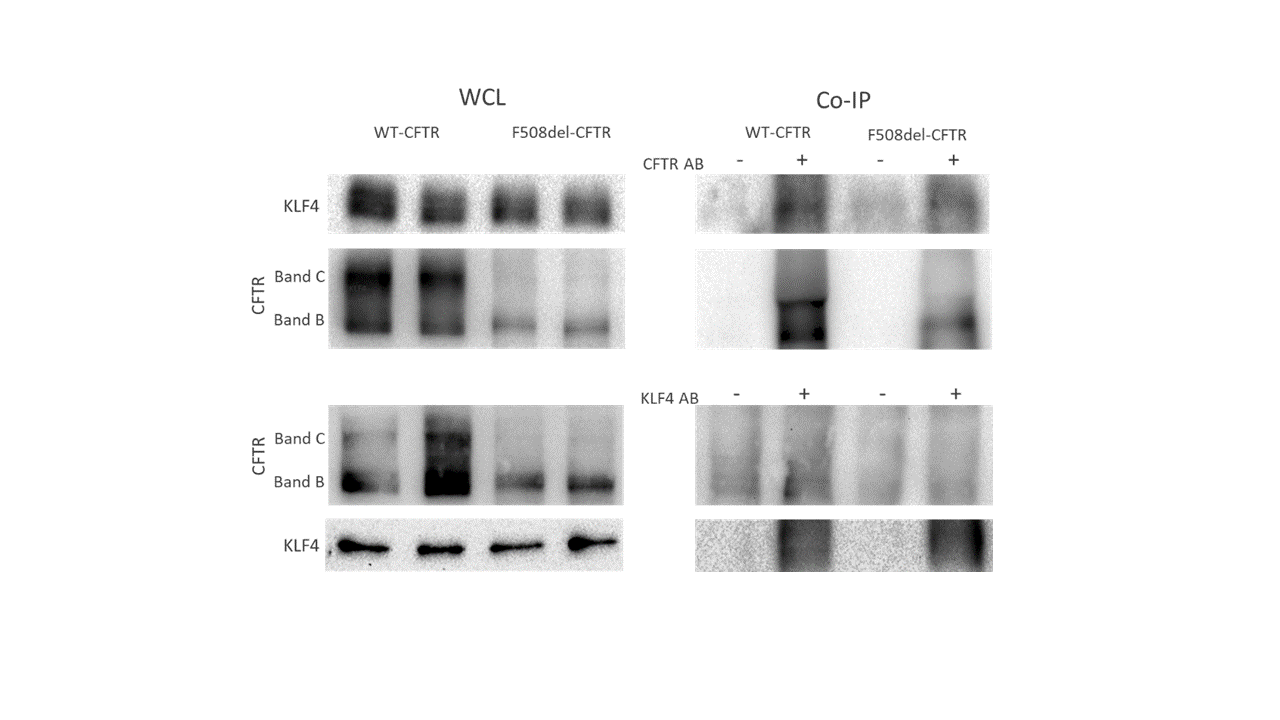

Supplement: Supplementary file 1 [file cells-09-01607-s001.zip › Slide4.PNG]

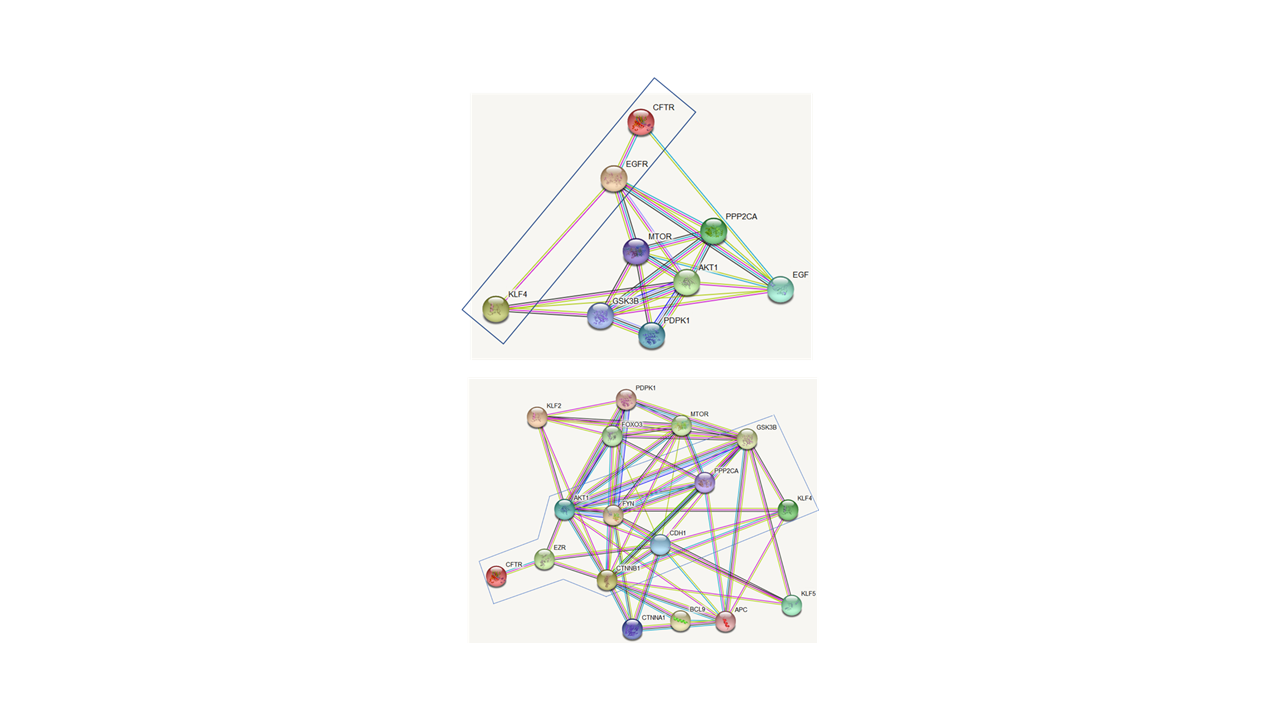

Supplement: Supplementary file 1 [file cells-09-01607-s001.zip › Slide5.PNG]
